# The Hippo signaling pathway provides novel anti-cancer drug targets

**DOI:** 10.18632/oncotarget.14306

**Published:** 2016-12-27

**Authors:** June Sung Bae, Sun Mi Kim, Ho Lee

**Affiliations:** ^1^ Biomolecular Function Research Branch, National Cancer Center, Goyang 10408, Republic of Korea; ^2^ Graduate School of Cancer Science and Policy, National Cancer Center, Goyang 10408, Republic of Korea

**Keywords:** Hippo, anti-cancer target, tumor suppressive pathway, YAP, TAZ

## Abstract

The Hippo signaling pathway plays a crucial role in cell proliferation, apoptosis, differentiation, and development. Major effectors of the Hippo signaling pathway include the transcriptional co-activators Yes-associated protein 1 (YAP) and WW domain-containing transcription regulator protein 1 (TAZ). The transcriptional activities of YAP and TAZ are affected by interactions with proteins from many diverse signaling pathways as well as responses to the external environment. High YAP and TAZ activity has been observed in many cancer types, and functional dysregulation of Hippo signaling enhances the oncogenic properties of YAP and TAZ and promotes cancer development. Many biological elements, including mechanical strain on the cell, cell polarity/adhesion molecules, other signaling pathways (e.g., G-protein-coupled receptor, epidermal growth factor receptor, Wnt, Notch, and transforming growth factor β/bone morphogenic protein), and cellular metabolic status, can promote oncogenesis through synergistic association with components of the Hippo signaling pathway. Here, we review the signaling networks that interact with the Hippo signaling pathway and discuss the potential of using drugs that inhibit YAP and TAZ activity for cancer therapy.

## INTRODUCTION

**Figure 1:**
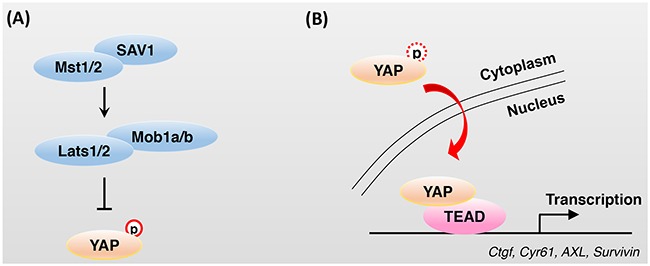
Regulation of YAP activity by Hippo core components **A**. The phosphorylation cascades of Hippo core components reduce the activation of the transcriptional co-activator YAP. Phosphorylated YAP is sequestered in the cytoplasm by 14-3-3 and recruits SCFβ-TrCP E3 ubiquitin ligase, which ultimately leads to YAP degradation. **B**. Impaired or attenuated activity of Hippo core components results in the dephosphorylation of YAP and translocation of YAP from the cytoplasm to the nucleus. In the nucleus, YAP cannot bind to DNA directly and TEAD family transcription factors, which are characterized by the presence of a TEA/ATTS DNA-binding domain, are key partners of YAP for DNA binding and transcriptional activation.

The Hippo signaling pathway plays a crucial role in cell proliferation, apoptosis, differentiation, and development. The phosphorylation cascades of Hippo core components (Hpo, Sav, Wts, and Mats in *Drosophila* and Mst1/2, Sav1, Lats1/2, and Mob1a/b in mammals) inhibit the activation of transcriptional co-activators Yorkie (Yki), YAP, and TAZ (Figure 1A). YAP and TAZ are major effectors of the Hippo signaling pathway. They function as transcription factors along with TEAD (TEA domain family member) in the nucleus, which increases expression of such target genes as *Ctgf*, *Cyr61*, *AXL*, and *Survivin* (Figure 1B). The phosphorylation of YAP and TAZ and activation of Lats kinase are regulated by multiple mechanisms. Many biological pathways and factors have been shown to affect the activity of the Hippo signaling pathway beyond the simple phosphorylation of YAP and TAZ by core components. We review the history and current understanding of the function and regulation of the Hippo signaling pathway and discuss some unresolved issues.

## BRIEF HISTORY OF THE HIPPO SIGNALING PATHWAY

Two decades ago, loss of the Warts (Wts) gene in *Drosophila* was shown to cause dramatic cell overproliferation and various developmental defects [1, 2]. Following this report, some groups showed that defects of the Salvador (Sav) [3, 4], Hippo (Hpo) [5–9], and Mats [10] genes resulted in an increase in tissue growth and impairment of apoptosis. All of these signaling molecules are directly involved in the Hippo signaling pathway, which depends on a phosphorylation cascade (Figure 1A). Yki was identified as a transcriptional co-activator and downstream effector of the Hippo signaling pathway in *Drosophila* [11]. Subsequent studies identified mammalian orthologs of pathway components and confirmed that this pathway is well conserved in mammals.

Because Yki, YAP, and TAZ cannot bind to DNA, they need to bind to another transcription factor that interacts with DNA directly. In *Drosophila*, Scalloped (Sd) binding to Yki increases the transcriptional activity of Sd [12]. Sd belongs to a family of evolutionarily conserved proteins that are characterized by the presence of a TEA/ATTS (Transcriptional Enhancer Activator/AbaA, TEC1 p, TEF-1 Sequence) DNA-binding domain. The mammalian orthologs of Sd, TEAD family transcription factors (TEAD1-4), are key YAP interaction partners (Figure 1B). TEAD or Sd bind specifically to a consensus motif (GTIIC and Sph sequence, TGGAATGT or ACATTCCA) and activate transcription *in vivo* [13]. While expression of TEAD or YAP causes marked cell-cycle progression and inhibits differentiation in neural progenitor cells, their loss of function results in an increase in apoptosis [12, 14, 15]. TEAD-binding-deficient YAP (S94A mutant) mimics YAP knockout phenotypes in the skin and heart [16, 17].

In mammals, the five consensus HXRXXS motifs in YAP (S61, S109, S127, S164, and S381) are phosphorylated by Lats kinase. Although all of these are phosphorylated *in vivo*, S127 and S381 are regarded as the critical sites related to increased oncogenic activity. S127 phosphorylation results in 14-3-3 binding and cytoplasmic sequestration. S381 phosphorylation induces the subsequent phosphorylation of S384 and S387 by CK1δ/ε. These phosphorylated phosphodegrons recruit the SCF^beta-TrCP^ E3 ubiquitin ligase, which ultimately leads to YAP degradation [18].

## RECENT REPORTS ON THE HIPPO SIGNALING PATHWAY

Although the immediate mechanism of regulation of YAP and TAZ by Hippo core components has been demonstrated, several proteins have recently been found to regulate Hippo core components in less direct fashion, bypassing the linear kinase modules to activate YAP and TAZ. These studies have shown that mechanical cues, cell polarity/adhesion molecules, other signaling pathways, and cellular metabolic status can regulate YAP and TAZ activity.

### Cytoskeleton modulators

The growth of normal cells in culture systems halts when they physically encounter other cells. YAP is inhibited by cell-cell contact (i.e., a high cell density) through Hippo signaling pathway activation, and overexpression of YAP can overcome cell-contact inhibition and promote cell proliferation [19].

**Figure 2:**
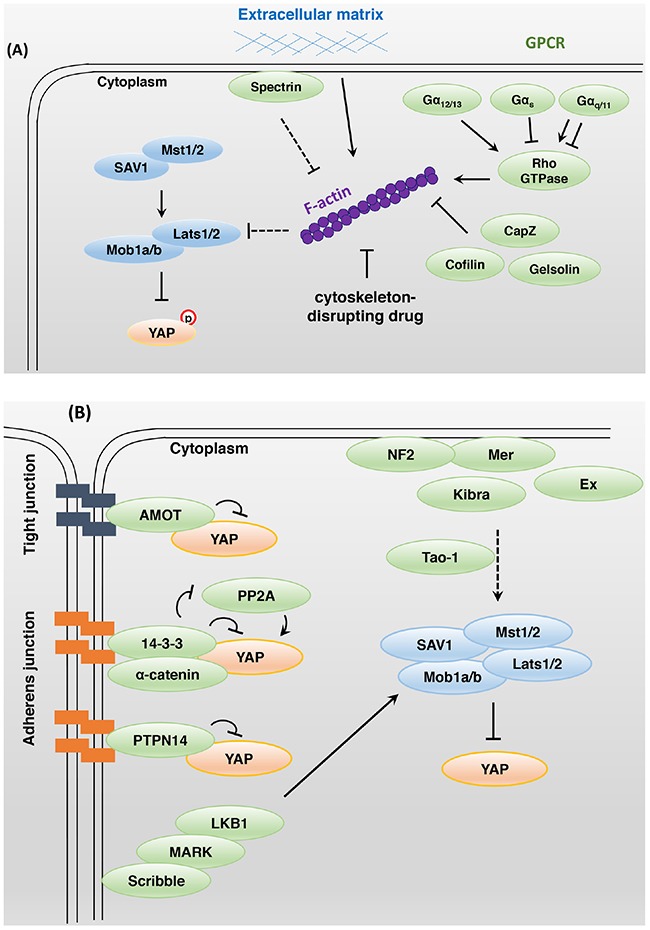

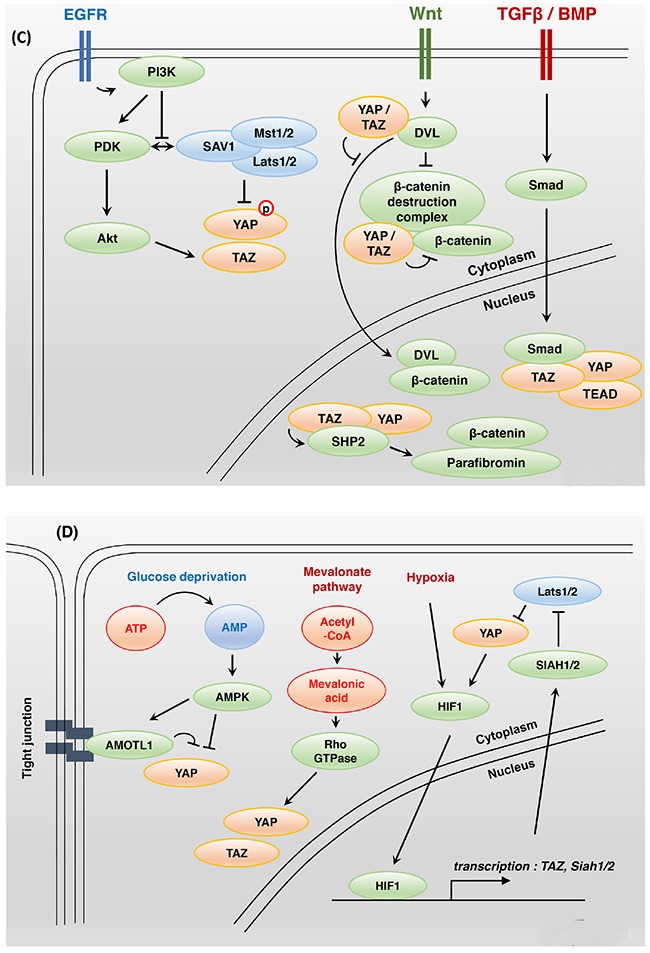
Bi-directional interactions between Hippo and other signaling pathways **A**. Modulation of the actin cytoskeleton affects YAP activity. Extracellular matrix stiffness, cell geometry, cell-cell contacts, and F-actin-modulated proteins regulate YAP and TAZ activity. Rho family small GTPases play a key role in actin cytoskeleton organization. LPA- and S1P-activated Gα_12/13_-protein-coupled receptors inhibit Lats1/2 kinase activity, whereas glucagon- and epinephrine-activated Gα_s_-protein-coupled receptors stimulate Lats1/2 kinase activity. Gα_q/11_ protein can either activate or inhibit YAP activity. **B**. In mammals, apical domain protein NF2 and Kibra act as upstream regulators of Hippo core components. To activate the Hippo signaling pathway, NF2 and Kibra may require Tao-1 kinase activity. The junctional proteins AMOT, 14-3-3, and PTPN14 interact with YAP directly and inhibit its activity. The basolateral domain protein Scribble acts downstream of the tumor suppressor LKB1 and MARK to regulate the Hippo signaling pathway. **C**. PDK1 associates with Mst and Lats kinase through Sav, and these complexes induce YAP-S127 phosphorylation. Epidermal growth factor-mediated PI3K activation triggers the dissociation of PDK1 and Hippo core components that fail to promote YAP phosphorylation. Nuclear YAP and TAZ promote SHP2-dependent dephosphorylation of parafibromin. The phosphorylated parafibromin interacts with β-catenin and activates Wnt target gene transcription. However, cytoplasmic YAP and TAZ inhibit the nuclear accumulation of β-catenin as integral components of the Wnt signaling pathway. TAZ, YAP, SMAD2/3, and TEAD physically interact in the nucleus and share a transcriptional program. **D**. Glucose deprivation leads to AMOTL1 phosphorylation by AMPK. Phosphorylated AMOTL1 is more stabilized and promotes YAP-S127 phosphorylation and nuclear exclusion. AMPK also inhibits the transcriptional activity of YAP through direct phosphorylation. The mevalonate pathway can promote Rho GTPase activity through geranylgeranylation, thus enhancing YAP and TAZ activity. Under hypoxic conditions, a positive feedback loop may be established to enhance HIF1, YAP, and TAZ activity.

The rigidity and cell geometry of the extracellular matrix (ECM) regulate YAP and TAZ activity (Figure 2A). While cells cultured on rigid hydrogels exhibit high YAP and TAZ activity with a broader cell-ECM contact area, culture on soft matrices inhibits YAP and TAZ activity resulting in a smaller cell-ECM contact area [20]. These results suggest that YAP and TAZ sense external mechanical cues and regulate cell proliferation based on proper cellular microenvironments. Cell attachment or detachment to the ECM leads to dramatic actin and microtubule cytoskeleton reorganization and YAP dephosphorylation or phosphorylation, respectively. The actin cytoskeleton-disrupting drugs latrunculin B and cytochalasin D inhibit YAP activation in response to cell attachment to the ECM (Figure 2A). Disruption of the actin cytoskeleton induces Lats kinase activity through activation of protein kinase A (PKA) [21]. In *Drosophila*, extra F-actin formation through the loss of capping proteins led to overgrowth in imaginal discs through the inhibition of Hippo signaling activity [22, 23]. In mammals, depletion of the actin-destabilizing proteins Cofilin, CapZ, and Gelsolin increases F-actin stability and YAP/TAZ activity, even at high cell density [24].

Rho family small guanosine triphosphatases (GTPases) play a key role in actin cytoskeleton organization (Figure 2A). The specific Rho GTPase inhibitor botulinum toxin C3 blocked YAP activation in response to cell attachment. Moreover, the G-protein-coupled receptor (GPCR) ligands lysophosphatidic acid and sphingosine 1-phosphophate inhibited Lats1/2 kinase through Gα_12/13_-protein-coupled receptors. In contrast, glucagon and epinephrine, which stimulate Gα_s_-protein-coupled receptors, activated Lats1/2 [25, 26]. Depletion of Rho GTPase activator AKAP13 attenuates YAP and TAZ activity by inhibiting organization of the actin cytoskeleton, which occurs independently of Lats activity in human embryonic stem cells [27].

Spectrin is a cytoskeletal protein found on the intracellular side of the plasma membrane and involved in cytoskeletal tension. Spectrin acts as a tumor suppressor and upstream regulator of the Hippo signaling pathway in *Drosophila*. Spectrin downregulation led to the phosphorylation of regulatory light chain of myosin II by Rho-associated protein kinase, resulting in the generation of contractile tension and Yki activation [28, 29].

Mechanical force appears to change cytoskeletal organization in a context-dependent manner and to modulate YAP activity. In *Drosophila*, Ajuba LIM protein binds to α-catenin and Wts at adherens junctions under conditions of high cytoskeletal tension and then inhibits Wts activity [30]. Mechanical stimuli can also activate c-Jun N-terminal kinase (JNK), leading to a JNK-dependent increase in LIMD1, which then binds and inhibits Lats1 kinase [31].

### Cell polarity/adhesion molecules

In *Drosophila*, Merlin (Mer), Expanded (Ex), Kibra, and Crumbs interact with the cytoskeleton at the apical domain of the cell. The FERM domain proteins Mer and Ex control proliferation and apoptosis through Hippo signaling activity [32]. The WW domain protein Kibra forms a complex with Mer and Ex and acts upstream of the Hippo core components to activate Hippo signaling in *Drosophila* [33–35] (Figure 2B). The apical transmembrane protein Crumbs is important for apical-basal polarity and binds with Ex to induce proper Hippo signaling activity [36–38]. The connection between Mer/Ex/Kibra and the Hippo signaling pathway may be needed for Tao-1 kinase activity. Tao-1 phosphorylates Hpo at Thr195 in *Drosophila* and Mst in mammals [39, 40]. Another report showed that Mer and neurofibromatosis type II (NF2; mammalian ortholog) anchor Wts and Lats to the plasma membrane, in turn promoting Wts and Lats phosphorylation by Hpo and Mst in an actin-mediated manner [41]. The immunoglobulin domain-containing cell adhesion molecule Echinoid (Ed) interacts with and stabilizes Sav at adherens junctions to activate Hpo [42]. Loss of Ed results in tissue overgrowth via high Yki activity in *Drosophila*.

In mammals, angiomotin (AMOT) family proteins inhibit YAP and TAZ activity via direct binding at tight junctions independently of YAP and TAZ phosphorylation status [43–45] (Figure 2B). α-Catenin is involved in tumor suppression and cell density sensing. This protein negatively regulates YAP by forming a complex with 14-3-3 and inhibiting the access of PP2A phosphatase for YAP dephosphorylation at adherens junctions [16]. Non-receptor-type protein tyrosine phosphatase 14 (PTPN14) is located at adherens junctions and binds to YAP. Under high cell density conditions, YAP is sequestered and inhibited by PTPN14 in the cytoplasm [46–48]. Scribble is localized at the basolateral domain of the cell membrane and where it suppresses tumorigenesis by forming a complex with Mst1/2, Lats1/2, and TAZ and thus inhibiting TAZ activity [49]. Scribble also acts downstream of the tumor suppressor LKB1 and microtubule affinity-regulating kinase (MARK) to activate the Hippo signaling pathway [50].

Although several lines of evidence indicate that the modulation of cell polarity/adhesion molecules influences Hippo core components and YAP and TAZ activity, their relationship may be mostly indirect. Additional studies are required to define the link between cell polarity/adhesion molecules and the Hippo signaling pathway.

### Other signaling pathways that interact with the Hippo signaling pathway

The Hippo signaling pathway is integrated upstream or downstream of other biological processes. The prominent signaling pathways related to the Hippo signaling pathway are GPCR, epidermal growth factor (EGF), bone morphogenetic protein (BMP)/transforming growth factor β (TGFβ), Wnt, and Notch signaling pathways (Figure 2A and 2C).

GPCR signaling has been implicated in almost every aspect of the physiological regulation of Hippo signaling (Figure 2A). As mentioned above, the activation of Gα_12/13_ stimulates YAP and TAZ activity by inhibiting Lats1/2 kinase [26, 51], and the activation of Gα_s_ activates Lats1/2 kinase activity [26]. Cyclic adenosine monophosphate (cAMP), a second messenger of Gα_s_, activates protein kinase A (PKA). Activated PKA activates Lats kinase by inhibition of Rho GTPases [21, 52]. Gα_q/11_ can activate protein kinase C (PKC), which can either activate or inhibit YAP activity, dependent on the isoforms of PKC [53].

Treatment with an EGF ligand induces PI3-kinase (PI3K)–phosphoinositide-dependent kinase (PDK1) axis activation and inhibits Hippo core components independently of AKT (Figure 2C). In confluent or serum-starved cells, PDK1 binds to Mst and Lats kinase through Sav, inducing YAP S127 phosphorylation. Epidermal growth factor-mediated PI3K activation triggers the dissociation of PDK1 and Hippo core components that fail to promote YAP phosphorylation [54]. EGFR signaling also promotes Ajuba phosphorylation through the Ras–mitogen-activated protein kinase pathway. Phosphorylated Ajuba binds to Lats and Sav and inhibits Hippo core component activity [55]. PI3K activation promotes TAZ stabilization, which is dependent on AKT [56]. The EGFR ligands amphiregulin (AREG) [57] and epiregulin (EREG) [58] may be transcriptional or downstream targets of YAP and TAZ. These findings suggest a positive feedback loop and intimate crosstalk between the EGF and Hippo signaling pathways.

TGFβ and BMP signaling acts as a ligand-induced transcriptional activator or repressor of responsive target genes that act through the SMAD (Sma and Mad-related family) protein complex [59] (Figure 2C). TGFβ stimulates TAZ to bind heteromeric Smad2/3-4 complexes, and the loss of TAZ results in a failure of Smad2/3-4 complexes to accumulate in the nucleus [60]. TAZ, YAP, TGFβ-activated Smad2/3, and TEAD physically bind to each other in the nucleus, and a genome-wide analysis revealed that they share a pro-tumorigenic transcriptional program [61]. YAP, TAZ, TEAD, Smad2/3, and Oct4 complexes suppress the transcription of a mesendodermal marker that inhibits differentiation in human embryonic stem cells [62]. YAP and TAZ activate TGFβ and Smad signaling in Mob1-deficient mouse liver, and these mice develop liver cancer, combined hepatocellular and cholangiocarcinomas, and intrahepatic cholangiocellular carcinomas [63]. Activated YAP/TAZ-TEAD bind the Tgfβ2 locus and increase transcription levels of Tgfβ2 in Lats1/2-deficient hepatoblasts and embryonic liver. Loss of Lats kinase activity promotes a lineage specification from hepatoblast to biliary epithelial cell but not to hepatocyte differentiation [64]. YAP also supports BMP–Smad1/5/8-dependent transcription. Receptor-activated Smad1 is phosphorylated by CDK8/9, which promotes Smad transcription action through the recruitment of YAP to the phosphorylated linker sites [65]. BMP4, a TGFβ superfamily growth factor, is a TAZ-dependent transcriptional target that promotes Smad1/5/8-mediated signaling in mammary epithelial cells [66].

Hippo and Wnt signaling reciprocally regulate each other's activity through a variety of mechanisms (Figure 2C). First, nuclear YAP and TAZ promote the nuclear accumulation of β-catenin, which in turn activates Wnt signaling. SHP2, a ubiquitously expressed protein tyrosine phosphatase, physically binds to YAP and TAZ. YAP and TAZ stimulate the nuclear translocation of SHP2, which in turn dephosphorylates parafibromin. The dephosphorylated parafibromin–β-catenin complex activates Wnt target gene transcription [67]. Second, cytoplasmic YAP and TAZ that are displaced from the nucleus by phosphorylation events are integral components of the Wnt destruction complex (Axin, GSK3, and β-TrCP) and promote the proteasomal degradation of β-catenin in the absence of Wnt activation [68–70]. Adenomatous polyposis coli (APC) is known as a negative regulator of β-catenin and important as a scaffold protein that activates Lats and Sav1. GSK3β and Axin2 are needed to facilitate Hippo signaling [71]. Cytoplasmic YAP and TAZ interact with Disheveled (DVL) and block DVL-dependent Wnt transcriptional activity [72]. Third, alternative Rho GTPase-dependent Wnt signaling can activate YAP and TAZ activity by inhibiting Lats kinase activity. Among the target genes of YAP and TAZ are secreted canonical Wnt inhibitor genes that inhibit the expression of β-catenin/TCF target genes [73]. Crosstalk between the Wnt and Hippo signaling pathways in intestinal tissue is clear, but more studies may be needed to verify whether the correlation between them is positive or negative and whether such a correlation exists in other organs or tissues beyond the intestine.

YAP/TEAD complex directly regulates the transcription of Jag1 [74] and Notch2 [75, 76]. Other Notch components and Notch target genes are elevated by YAP activation in mouse liver [76]. During ductal reactions and biliary regeneration in liver, the activation of Jag1-Notch2-mediated Notch signaling is important for the differentiation of hepatic progenitor cells into biliary-specific cells [77, 78]. YAP also promotes proliferation but prevents differentiation in satellite cells (myogenic stem cells in skeletal muscle) via Notch [79, 80]. However, Mob1-deficient mice exhibit elevated Jagged1 but not Notch2 or Hes1 despite YAP activation [63]. It is still unclear whether YAP transcriptional activity directly leads to Notch signaling activation in diverse situations.

### Cellular metabolic status affects Hippo signaling

In response to energy deprivation, cell growth and proliferation are inhibited, and YAP and TAZ activity decreases (Figure 2D). Energy stress induced by 2-deoxy-D-glucose, 5-aminoimidazole-4-carboxamide ribonucleotide and Metformin activates AMP-activated protein kinase (AMPK), which directly phosphorylates angiomotin-like 1 (AMOTL1). Phosphorylated AMOTL1 promotes YAP-S127 phosphorylation and nuclear exclusion in a Lats kinase-dependent manner [81]. AMPK also inhibits the transcriptional activity of YAP-TEAD by directly phosphorylating YAP S61 [82] and the TEAD binding motif YAP S94 [83]. High YAP transcriptional activity enhances glucose-transporter 3 (GLUT3) transcription, promoting glycolysis, and YAP and GLUT3 expression is positively correlated in human cancers [82]. A sufficient glucose supply can activate YAP and TAZ activity and phosphofructokinase 1 (PFK1), a glycolytic enzyme, mediates glucose-induced YAP- and TAZ-TEAD interactions [84].

Under hypoxic conditions, hypoxia-inducible factor 1 (HIF1) is rapidly accumulated, and the transcriptional activity of HIF1 is elevated in response to changes in available oxygen in the cellular environment. The hypoxia-induced HIF1 transcriptional target genes include TAZ and ubiquitin ligase SIAH1/2, which is required for the ubiquitination and degradation of Lats2 kinase [85, 86] (Figure 2D). YAP also binds to and stabilizes HIF1 [86], and a positive feedback loop may be established to enhance HIF1 and YAP/TAZ activity under hypoxic conditions.

The mevalonate pathway produces numerous essential metabolic biomolecules, such as cholesterol, steroid hormones, and vitamin K from acetyl-coenzyme A (CoA) [87]. An inhibitor of the enzyme 3-hydroxyl-3-methylglutaryl-CoA (HMG-CoA) reductase converts HMG-CoA to mevalonic acid, causing marked cytoplasmic accumulation of YAP and TAZ (Figure 2D). Mevalonic acid is a precursor of geranylgeranyl pyrophosphate, which promotes G_βγ_ protein and Rho GTPase activity though geranylgeranylation, enhancing YAP and TAZ activity [88, 89]. Mevalonate-mediated YAP and TAZ activation increases the expression of the receptor for hyaluronan-mediated motility (RHAMM), contributing to the tumorigenesis and metastasis of many tumors [90].

### Hippo signaling and cancer

Elevated YAP and TAZ activity induce hyperplasia, dysplasia, and tumors in a number of mouse models. YAP-inducible transgenic mice show irregular expansion in liver tissue and undifferentiated intestinal progenitor cells [91]. YAP induces transcription of cell-cycle regulator Cyclin E and IAP / BCL2 family proteins in *Drosophila*, and Cyclin D and Bcl_XL_ expression levels are correlated with YAP expression levels in human colorectal cancers [91, 92]. YAP also assures cells ongoing proliferation after radiation. Radiation resistance conferred by YAP induces genomic instability and complicates radiation therapy for cancer patients [93]. Thus, YAP activation confers potent cell proliferation capacity and resistance to apoptosis which can eventually lead to cancer development. In addition, TAZ stimulates the epithelial-to-mesenchymal transition through transcriptional activation of ZEB1/2 [94, 95].

Deletion of Hippo core component genes can also cause hyperplasia or expansion of specific cell types. In the liver-specific Lats1/2-knockout mouse, liver is filled with highly proliferated immature biliary epithelial cells [64]. Deletion of Sav1, Mst1/2, or Lats2 promotes cardiomyocyte proliferation at the embryonic stage [96]. Mst1/2 deletion causes hepatocellular carcinoma, cholangiocarcinoma or bile duct hamartoma [97–99]. Hepatocyte- and intestinal epithelial cell-specific deletion of Sav1 leads to increase of hepatic progenitor cells and intestinal progenitor cells, respectively [100, 101]. Various types of tumor arise in hypomorphic Mob1 mouse [102], and phenotypes of liver-specific Mob1-deficient mice are similar to Lats1/2-knockout liver [63]. Because, in many cases, dysregulation of the Hippo signaling pathway and increased YAP/TAZ activity induce expansion of tissue-specific stem/progenitor cells which eventually leads to development of cancer, uncovering the physiological roles of the Hippo signaling pathway and YAP/TAZ in a tissue homeostasis and cancer stem cells would be helpful for cancer therapy.

The correlation between Hippo signaling and cancer is not the same in all tissues or cells. For example, the epidermis of mice with skin-specific deletion of Mst1/2 or Lats1/2 shows no abnormality and YAP-S127 phosphorylation is not increased [16]. Also, no kidney defects or YAP activation are seen after deletion of Mst1/2 or Sav1 [103]. Thus, YAP activity is not always elevated by restriction of Hippo core components. Meanwhile, there is a case in which oncogenic phenotype does not occur in spite of the activation of YAP. Intestinal epithelial cell-specific YAP transgenic expression leads to rapid loss of intestinal crypts and inhibition of Wnt-mediated intestinal regeneration [72]. These results suggest that the Hippo signaling pathway and YAP/TAZ have different tissue- or cell-type-specific physiological roles.

Although the dysregulation of YAP has been reported in many types of cancer, little is known about the contributions of mutations of Hippo signaling pathway genes. Data from the Catalogue of Somatic Mutation in Cancer (COSMIC) census show that Hippo pathway genes, with the exception of NF2 and TAZ, are not cancer-related genes [104]. Although recent studies have reported that some mutations of YAP, Lats1/2, GPCRs, NF2, and TAZ are related to the development of cancer [105], it is still unclear why the mutations in Hippo signaling pathway genes are so rare, when considering that YAP and TAZ activation is so frequently observed in a broad range of human cancers. One possibility is that YAP and TAZ activation is affected by other signaling pathways (e.g., Wnt [106], TGFβ-BMP [107], Notch [108], EGFR [109], and GPCR [110]) that frequently harbor oncogenic mutations. The activation of these other signaling pathways is correlated with the immunohistochemical detection of nuclear YAP and TAZ in tumor tissues [63, 74, 111–113]. In addition, mutations of Hippo signaling pathway genes and crosstalk with other aberrant signaling pathways may result in the development of cancer. Further investigation is required to define the major oncogenic sources that cause YAP and TAZ activation in various tumor types.

## INHIBITORS OF HIPPO SIGNALING AS ANTI-CANCER DRUG CANDIDATES

As discussed above, the Hippo signaling pathway is interconnected with other biological processes that promote tumor formation and development. YAP and TAZ activity is high in many cancer types, which suggests that Hippo signaling components are potential targets for cancer therapy. The Hippo core kinases Mst1/2 and Lats1/2 are the most attractive therapeutic targets. However, Mst1/2 and Lats1/2 kinases are tumor suppressive, and their activity is low in many cancer types, unlike most oncogenic kinases, such as Src, Abl, Raf, and Akt, which are activated by mutations and involved in tumorigenesis [114]. Therefore, agonists that can enhance the Hippo core kinase activity are required for developing anti-cancer drugs.

Recently, YAP was identified as a key survival input that mediates resistance by acting parallel to other known pathways in cancer progression. YAP silencing enhanced the response to RAF and MEK inhibitor in a wide spectrum of BRAF-mutated cancer cell lines, and patients who have BRAF-mutated tumors with lower YAP expression respond better to treatment with RAF and MEK inhibitor [115, 116]. Increased actin cytoskeleton tension is accompanied by activation of YAP/TAZ in BRAF-mutated melanoma which acquires resistance to RAF and MEK inhibitor. Suppression of actin cytoskeleton assembly and tension inhibits both YAP/TAZ activation and BRAF inhibitor resistance [117]. In addition, colon cancer cell lines with elevated YAP activity are resistant to 5-fluorouracil[118], and breast cancer cell lines expressing activated TAZ are more resistant to doxorubicin/paclitaxel [49] and taxol treatment [119]. However, the mechanism by which chemotherapy elevates YAP/TAZ activity is not clear, and further investigation is needed to address how activated YAP/TAZ confers chemotherapy resistance to cells as well as to clarify the oncogenic properties of YAP/TAZ.

The direct inhibition of YAP and TAZ oncoproteins is also a therapeutic target for anti-cancer treatment. Heterozygous and homozygous deletion of YAP dramatically suppresses tumorigenesis in LKB1-, Mob1-, and NF2-deficient liver [50, 63, 120], Mst1/2-deficient phenotypes in the small intestine and colon [121], and the early progression to pancreatic ductal adenocarcinoma in a Kras (G12D) cancer model [122]. The function of transcriptional co-activator YAP/TAZ are primarily mediated by other transcription factors, such as TEADs, SMADs, and RUNX. Thus, disrupting the interaction with its partner transcription factor could inhibit YAP/TAZ's oncogenic activity. The structural information of YAP-TEAD complex is well established [123–125]. YAP has an N-terminal TEAD-binding domain, and point mutations of M89A, R89A, L91A, S94A, F95A and F96A of the twisted-coil region of YAP strongly diminished YAP-TEAD activity *in vitro* [123]. Lentiviral expression of YAP fragment 86-100, which weakly interacts with TEAD, has no significant effect on the YAP-TEAD activity [123]. The TDU domains of VGLL4, a natural antagonist of YAP, compete with YAP for TEAD4 binding. Inhibitor peptide derived from the TDU domain potently suppresses tumor growth *in vitro* and *in vivo* [126]. Further investigation will be needed to design YAP inhibitors based on the YAP-TEAD structure to selectively inhibit YAP-induced tumorigenesis.

In high-throughput screening, Verteporfin, a small molecule that belongs to the porphyrin family, was identified as an inhibitor of YAP-TEAD interactions (Table 1). Treatment with Verteporfin inhibited YAP-induced liver overgrowth in NF2/Merlin inactivation [127]. Many researchers have discovered numerous small molecules that can regulate Hippo and YAP upstream components through the GPCR, Rho, cAMP, F-actin, EGFR, and mevalonate pathways (Table 1). These small molecules inhibit the transcriptional activation by YAP and TAZ in mice and cultured cancer cell systems and may be applicable as anti-cancer drug candidates.

**Table 1:** Inhibitors of Hippo pathway

Inhibitors/agonist	Target	Effects	Refs.
Verteporfin	YAP	Binding to YAP to inhibit the interaction of YAP with TEAD and its transcriptional activity Suppressing liver overgrowth caused by YAP overexpression or NF2 inactivation	[127]
Dasatinib	YES1	Inhibiting the tyrosine 357 phosphorylation of YAP Inhibiting interaction of YES1, YAP, and β-catenin complex independent of YES1 kinase activity Inhibition of gut formation to a similar extent as the YAP or YES1 knockdown Impeding β-catenin-dependent proliferation of colon cancer cell lines.	[111]
Y27632	ROCK		[20, 22]
Blebbistatin	non-muscle myosin	Inhibition of YAP/TAZ nuclear localization and transcriptional activity	
Latrunculin A	F-actin		
Latrunculin B	F-actin	Inhibition of nuclear YAP localization via enhanced LATS activity	[25]
Cytochalasin D	F-actin	Inhibits nuclear YAP localization via enhanced LATS activity and reduces TEAD-dependent transcriptional activity	[22, 23, 25]
Botulinum toxin C3	Rho	Elevates phosphorylation of YAP/TAZ and blocks LPA- and S1P-induced YAP/TAZ dephosphorylation Inhibits YAP/TAZ nuclear localization and transcriptional activity.	[20, 25]
Dihydrexidine	Dopamine receptor	Increase of YAP phosphorylation	[25]
Dobutamine	G protein-coupled β-adrenergic receptor agonist	Induction of cytoplasm accumulation of GFP-YAP and YAP S127 phosphorylation Inhibiting YAP-dependent transcriptional activity independent of Hippo core kinases	[128]
Rolipram			[52]
IbudilastIBMXTheophylline	Phosphor-diesterase 4, adenylyl cyclase	Induction of YAP/TAZ phosphorylation dependent on LATS 1/2 kinase activity Inhibiting YAP-dependent transcriptional activity	
Forskolin			
LY294002PDK1 inhibitor II	PI3K, PDK1	Blocking dissociation of PDK1 from Hippo core components in response to EGF treatment. Inhibiting YAP dephosphorylation, nuclear accumulation and transcriptional activity	[54]
StatinsGGTI-298Zoledronic acid	HMG-CoA reductaseGeranylgeranyl transferaseFarnesyl diphosphate synthase	Stimulation of hippo signaling via increasing phosphorylation level of Mst1/2 and Lats1 kinase Reduces of TAZ expression and enhances YAP S127 phosphorylation Blocking YAP nuclear localization and transcriptional activity Rescue of eye overgrowth induced by Yki overexpression in drosophila	[88–90]
Simvastatin	Receptor for RHAMM transcription.	Inhibiting YAP-TEAD binding to RHAMM (hyaluronan-mediated motility) promoter	[90]
IvermectinMilbemycin D	Unknown	Inhibiting YAP dephosphorylation, nuclear accumulation and transcriptional activity Reduction of hepatomegaly and accumulation of improper differentiated cells in Mob1-deficient mice liver	[63]

## DISCUSSION

Hippo core components and YAP and TAZ activity are regulated by a variety of mechanisms. A number of studies have reported that the Hippo signaling pathway is associated with cellular mechanical strain, cell polarity/adhesion molecules, other signaling pathways (e.g., GPCR, EGFR, Wnt, Notch, and TGFβ/BMP), and cellular metabolic status. Dysregulation of Hippo signaling generally results in aberrant YAP and TAZ transcriptional activity that enhances their oncogenic properties. High YAP and TAZ activity has been detected in many types of cancer, but genetic mutations of Hippo core components, YAP, or TAZ are rarely found. This implies that aberrant activation of the Hippo signaling pathway may be insufficient for cancer formation *in vivo*. Activation of YAP and TAZ may be triggered by other biological processes that are vulnerable to oncogenic mutations and increases in the transcription of genes that are related to oncogenic processes. Understanding the synergistic effects between the Hippo signaling pathway and other biological processes that result in tumorigenesis could facilitate the development of new anti-cancer drugs.
